# K-means-based heterogeneous tunneling data analysis method for evaluating rock mass parameters along a TBM tunnel

**DOI:** 10.1038/s41598-023-49033-0

**Published:** 2023-12-07

**Authors:** Ruirui Wang, Lingli Zhang

**Affiliations:** School of Civil Engineering, Shandong Jianzhu University, Jinan, China

**Keywords:** Civil engineering, Geology

## Abstract

Rapid and accurate judgment of the rock mass condition is the key to guaranteeing the safety and efficiency of tunnel boring machine (TBM) tunneling. This paper proposes a method for evaluating rock mass parameters based on K-means clustering, grouping tunneling areas according to the values of TBM tunneling parameters. A dataset including rock mass and TBM tunneling data is treated by logistic normalization and principal component analysis (PCA), and large volumes of tunneling data with different features are transformed into appropriate volumes of dimensionless data. K-means clustering is used, samples are grouped according to the values of tunneling data, and the specific ranges as defined by clustering are regarded as the unified evaluated results of each group. Based on the C1 part of the Pearl Delta water resources allocation project, 100 training samples and 30 testing samples were field-collected, and the proposed method was realized by the training samples and verified by the testing samples. The evaluation accuracies of uniaxial compressive strength (UCS), and joint frequency (Jf) were 90%, and 86.7% respectively, demonstrating that the evaluation had acceptable values, and the proposed method was greatly helpful for judging rock conditions.

## Introduction

The tunnel boring machine (TBM) is widely used in tunnel excavation, especially for long and deep tunnels, because of its high efficiency and safety. To improve safety and efficiency, methods for choosing reasonable operating parameters have become the object of much research. Sun et al. explored the changes of tunneling parameters and the working load of a multi-machine structure, and analyzed the rationality of each combination of operating parameters, providing a basis for determining optimal operating parameters^1^. Xue et al. investigated rock-breaking conditions under different cutter penetrations and spacings through linear cutting tests and determined the optimal combination of tunneling parameters under different compressive strengths^2^. Zhang et al. used data mining to establish a mapping between rock mass and TBM tunneling data, and developed an intelligent decision control system by analyzing the operation process of the TBM main driver^3^. Liu et al. analyzed the influence of cutterhead thrust on tunneling speed under rock conditions with different Q_TBM_ values, and established a TBM construction simulation model with single and double shields, which can be used as a reference for adjusting the cutterhead thrust^4^.

The above research has great reference value for guaranteeing the safety and efficiency of TBM tunneling. It is worth noting that optimizing TBM operation parameters based on this research requires the geological data as priori information. Therefore, it is important to develop a rapid and accurate method to acquire the rock mass condition. However, limited by the complex machine structure and narrow space of TBM, in-field rock mass parameter testing methods applicable to the surface might be difficult to use in this environment, and many researchers have studied appropriate rock mass parameter test methods. Nacimipour et al. developed the rock strength boring probe (RSBP) to detect geological data including uniaxial compressive strength and tensile strength, according to the measured scratch depth of the equipment on the rock surface^5^. Wang et al. developed the true triaxial rock drilling test system (TRD), which can be used to test rock mass parameters under multiple rock type conditions^6^. Goh et al. analyzed share wave velocity by spectral analysis of surface waves (SASW) to measure the rock quality designation (RQD)^7^. Kong et al.^8^ and Liu et al.^9^ used the point-load testing results to evaluate the rock compressive strength.

The above research gives an effective idea on the acquisition of geological data and rock mass condition in TBM tunnels. However, most of these methods spend time on testing or analysis process, which yields geological data with hysteresis, and it is difficult to acquire the data at the speed of TBM excavation. To solve this problem, it is feasible to build a mapping between real-time TBM tunneling data and rock mass parameters by statistical methods, including traditional regression and data mining, which have widely used to evaluate rock mass parameters and achieved acceptable application. However, two key problems limit the effect of predicting rock mass parameters. The first one is the differences of the volume and structure of rock mass parameters and TBM tunneling data, and the second one is the effect limitation of single prediction model.

First, rock mass and TBM tunneling data show significant differences in volume and structure. Hence a dataset including rock mass and TBM tunneling data is complex heterogeneous data, for which it is difficult to directly establish statistical rules. Limited by space and testing time, the geological conditions of a long tunnel section are always expressed by a single combination of rock mass data. TBM tunneling data have hundreds of features, which change continuously and are collected with high frequency. Therefore, in the same tunneling area, a single combination of rock mass data corresponds to high volume tunneling data with a number of features and time-series samples. To construct key-value datasets, in current methods, representative tunneling data are always selected according to artificial cognition, which may result in the missing of tunneling information. To solve this problem, an effective dimension reduction method should be used to simplify the structure of the tunneling data, so as to retain as much data information as few features as possible.

Second, TBM always faces with complex rock mass conditions, which might be included in field-collected data. However, the changing rules between tunneling data and rock mass parameters differ according to rock conditions, and a single mapping may not be suitable for different rock mass conditions. In most traditional researches, single rock-machine mapping is used to evaluating rock mass parameters. For example, Mikaeil et al.^10^, Hassanpur et al.^11^, Samaei et al.^12^, Nelson et al.^13^, Grima et al.^14^, and Entacher et al.^15^. proposed models to describe the changing rule between rock mass and TBM tunneling parameters, and Armaghani et al.^16^, Zare et al.^17,18^, Mahdeveri et al.^19^, Liu et al.^20^, Yagiz et al.^21^, and Minh et al.^22^ applied machine learning algorithms such as artificial neural networks, particle swarm optimization, fuzzy logic, and gene expression programs to the establishment of rock-machine mapping, with good results. On this basis, some researchers have categorized known rock conditions and established mappings according to these. For example, Gong et al.^23,24^ established multiple mappings between TBM penetration and the brittleness index, under different joint orientations, volumetric joint counts and uniaxial compressive strengths, and obtained good evaluation results, Liang et al.^25^ used K-means clustering to evaluate rock mass discontinuity attitude elements, and obtained good accuracy. Cui et al.^26^ proposed an improved clustering method based on differential evolution, which performed well at the identification of rock discontinuity. Kitzig et al.^27^, Saeidi et al.^28^, Red et al.^29^, Wang et al.^30^, Li et al.^31^, Li et al.^32^, Gao et al.^33^, Majdi et al.^34^, Fattahi^35^, and Bashari et al.^36^ also use clustering methods to group rock mass parameters and obtained acceptable results. The results have also shown that multiple mappings based on grouped rock mass conditions have higher focalization than single regression mapping and requires the added step of categorizing rock mass condition, which is always empirically conducted. The clustering algorithm in machine learning, as a more scientific classification method, brings a good reference for evaluating rock mass parameters.

This paper is aiming at proposing a method to evaluate rock mass parameters based on known TBM tunneling data, and making contributions to solve the above-mentioned two problems. In detail, this paper proposes the evaluation of rock mass parameters by analyzing complex heterogeneous tunneling parameters. In detail, Zero-mean, Logistic normalization and principal component analysis (PCA) method are conducted firstly to simplify the TBM tunneling data. On this basis, K-means clustering is used to group field-measured samples with similar tunneling data. The rock mass parameter ranges of groups are then recorded as the evaluated results of each group. The statistical results can also be used to evaluate the rock mass parameters of newly encountered conditions during TBM tunneling. The method is verified by the rock mass and TBM tunneling parameters with correspondence collected in field, and the evaluation results have acceptable rationality and well matched with the rock mass parameters. The results proved that the proposed method is effective and helpful for solving he volume and structure difference between tunneling and rock mass data and evaluating rock mass parameters with acceptable accuracy.

## Method

### Overview of method

Rock strength and integrity are two main kinds of geologic factor on TBM tunneling and rock broken. Among them, the rock strength partly determines the force required for rock breaking, while the rock integrity influences the formation efficiency the rock fragmentation, and further influences the rock broken efficiency. In this paper, uniaxial compressive strength (UCS) is used to characterize rock strength, and joint frequency (Jf) is used to characterize rock integrity, and both of them are used as the evaluating targets.

There are two main tasks in this paper. One is to divide the stratum condition into different groups according to tunneling data. In TBM tunneling, tunneling data, including more than 200 features, such as TBM thrust, and torque, is obtained by equipped acquirement system and regarded as known quantity, while the rock mass parameters are difficult to obtained and needed to evaluating as unknown quantity. This step aims to clustering the unknown quantity (rock mass parameters) into groups according to known quantity (tunneling parameters) as grouping standards. According to the grouping results, each group can be used to train independent mapping, and the results can be helpful for building multiple rock-machine mappings to improve the pertinence and generalization ability of them.

The other task is to evaluate the rock mass parameters according to the clustering results. Filed collected samples are clustered into several groups, samples in the same group always have similar rock parameter ranges. Therefore, the range expressed in percentiles can be used as the evaluation results for each group.

**Figure 1:**
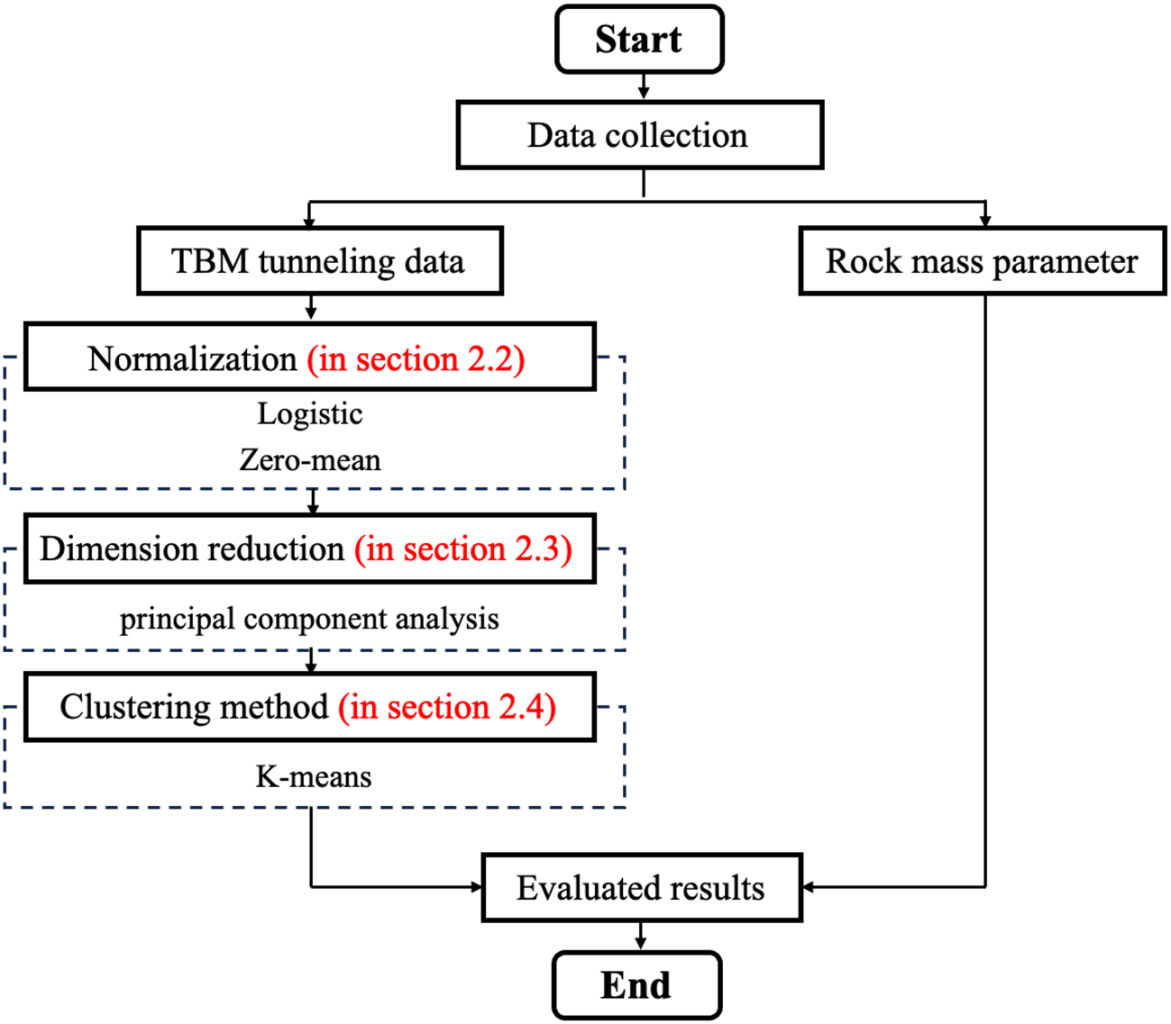
Flowchart of the proposed method.

Figure 1 is the flowchart of the proposed method. Before the method works, a field-collected data set should be prepared. The dataset should have sufficient samples, each of which includes tunneling parameters (as input) and rock mass parameters (as output) collected from the same tunneling location. For the complex tunneling data with high acquisition frequency, number of features, and different orders of magnitude, three key steps are used to simplify them, as discussed in "Normalization of rock mass and TBM tunneling parameters" to "Clustering and evaluation method based on k-means algorithm". Firstly, Zero-mean integrated with Logistic normalization is used balance the orders of magnitude of features of tunneling data (introduced in "Normalization of rock mass and TBM tunneling parameters"). Then, PCA is used to reduce the number of features and simplify the tunneling data (introduced in "Dimension reduction of TBM tunneling data based on principal component analysis"). Afterword, K-means is used to cluster samples into groups (introduced in "Clustering and evaluation method based on k-means algorithm"). On the basis of the clustering results, the rock mass parameters distribution of each group can be obtained, which is also regarded as the evaluating results of new-encountered rock condition (introduced in "Clustering and evaluation method based on k-means algorithm").

### Normalization of rock mass and TBM tunneling parameters

The aim of normalization is to transform the different features of in-field collected rock mass and TBM tunneling parameters to span a similar range, and to remove their dimensions. However, TBM tunneling data are complex, with more than 200 features with different dimensions and orders of magnitude. For example, data directly related to tunneling, such as thrust, and torque, usually has large positive value, and their magnitude is inconsistent. Data used to control the tunneling direction, such as horizontal and vertical angles, have both positive and negative value, representing the different directions. Some data such as the motor current of the cutterhead are always stable and close to a certain value, with small fluctuations.

Obviously, different methods to unify the dimension and order of magnitude, might lead to different results, and hence should be chosen cautiously according to the data range and distribution characteristics. A simple normalization method might not be suitable for such complex and varying data. Using Zero-mean or Logistic normalization may result in the influence of features with the value closed to 0 being suppressed. Therefore, we proposed a two-step method. The magnitude and dimensions of all TBM tunneling data are removed by Zero-mean normalization, and they then have the same range. Logistic normalization is then used to balance the fluctuation difference between features. Zero-mean and Logistic normalization are introduced as follows.

Zero-mean normalization

Usually, data that obey the standard normal distribution, i.e. with a mean of 0 and variance of 1, are considered to be suitable for clustering. The variable ${x}_{i}^{\prime}$, with mean µ and variance σ, is normalized as.

1 $${x}_{i}^{\prime}=\frac{{x}_{i}-\mu }{\sigma }.$$

As Eq. (1) shows, the values after Zero-mean normalization are between -− 1 and 1, which can make the original feature dimensionless and remove large value. However, the tunneling data, especially features closed to 0, may be easily influenced by abnormal outlies. Directly using Zero-mean normalization may result in most normal values still closed to 0, and their distribution rules are suppressed. For amplifying the influence of these feature, Logistic normalization needs to be used after Zero-mean normalization.

Logistic normalization

Logistic normalization is a nonlinear method, and is expressed as

2 $${x}_{i}^{\prime}=\frac{1}{1+{e}^{-x}}$$

As Eq. (2) shows, original positive data with larger absolute values become closer to 1, and original negative data become close to 0. Compared with feature with large original absolute value, feature closed to 0 has larger gradient. Through logistic normalization, the floating strength of some features near a constant is amplified, which reduces the negative influence of abnormal outlies and helps make full use of these features in subsequent data processing. If using Logistic normalization alone, the amplification effect may not enough because of the existence of features with high absolute values.

### Dimension reduction of TBM tunneling data based on principal component analysis

There are more than 200 features of TBM tunneling data which requires feature elimination, also known as dimension reduction. There are usually two solutions. One is to extract several key features according to relevance, or create several new features by mining or recursive methods. Considering the complexity of the TBM machine structure and the tunneling procedure, to directly reduce the number of features from 200 to less than 10 might be unsuitable because of the information lost. Therefore, we adopt a second method, using PCA to create new features.

The basic principle of PCA is to reorganize original variables to form new and independent variables through orthogonal transformation^37,38^. Take a dataset with ***m*** samples and ***n*** features as an example. The average values of each feature are normalized to zero, and the covariance between the handled features is calculated to form an n-dimensional covariance matrix,

3 $$C=\left[\begin{array}{ccc}cov\left({x}_{1},{x}_{1}\right)& \begin{array}{cc}cov\left({x}_{1},{x}_{2}\right)& \dots \end{array}& cov\left({x}_{1},{x}_{n}\right)\\ \begin{array}{c}cov\left({x}_{2},{x}_{1}\right)\\ \dots \end{array}& \begin{array}{cc}cov\left({x}_{2},{x}_{2}\right)& \dots \\ \dots & \dots \end{array}& \begin{array}{c}cov\left({x}_{2},{x}_{n}\right)\\ \dots \end{array}\\ cov\left({x}_{n},{x}_{1}\right)& \begin{array}{cc}cov\left({x}_{n},{x}_{2}\right)& \dots \end{array}& cov\left({x}_{n},{x}_{n}\right)\end{array}\right].$$

where *cov*(*x*_*i*_, *x*_*j*_) is the covariance between the *i*th and *j*th features. The greater the absolute value of the covariance, the greater the influence of the *i*th and *j*th feature on each other. A positive covariance means the two features increase or decrease together.

4 $$Cu=\lambda u.$$

As an n-dimensional matrix, *C* has *n* eigenvalues and corresponding eigenvectors *λ*_i_ and *u*_*i*_, respectively, including repeated values, i.e. the multiple solutions of Eq. (4). The absolute value of λi measures the amount of information of the handled feature included by the *i*th eigenvectors. Therefore, *r* eigenvalues with high average absolute values are selected, and the corresponding eigenvectors are regarded as the new and independent variables used in clustering. The value of *r* is a key factor in this procedure. If *r* is too small, this might lead to serious loss of feature information, while too large a value results in redundant variables and adds calculational burden. Usually, the *r* value is chosen according to the data used, as discussed in "Results".

### Clustering and evaluation method based on k-means algorithm

On the basis of the pretreated and refined data, K-means clustering is used to categorize tunneling areas according to their tunneling parameters. K-means has the advantage of fast calculation speed and good interpretability. A small number of samples is sufficient for a relatively stable stratum without sharp changes. Hence the rock mass dataset usually contains tens to hundreds of samples, and K-means performs sufficiently well for such a volume of data.

Before executing the K-means algorithm, the number of target groups *n* should be determined. On this basis, *n* samples are randomly selected as the center of each group, and recorded as *x*_*1*_, *x*_*2*_, …*x*_*n*_. The cluster center is the criteria for determining the group to which a sample belongs. Specifically, the sample belongs to the same group as its nearest cluster center. Each selected sample represents a group (*c*_*1*_, *c*_*2*_,…*c*_*n*_). The Euclidean distances between each sample and group center can be calculated. On this basis, each sample is grouped with the nearest group center, and the samples are divided in *n* groups, whose geometric centers can be calculated as

5 $${C}_{i}={\left(\frac{1}{{n}_{i}}\sum_{k=1}^{{n}_{i}}{x}_{i1k},\frac{1}{{n}_{i}}\sum_{k=1}^{{n}_{i}}{x}_{i2k},\dots ,\frac{1}{{n}_{i}}\sum_{k=1}^{{n}_{i}}{x}_{i{n}_{i}k} \right)}^{T}.$$

where *C*_*i*_ and *n*_*i*_ are respectively the geometric center and total number of samples of the *i*th group, and *x*_*ijk*_ is the value of the *j*th feature of the *k*th sample in the group. The geometric center of each group can then be calculated, and this is regarded as the new group center. The calculation of geometric centers and division of samples is performed iteratively, until the results are stable.

For a finished clustering result, silhouette coefficients (SC) are always used to evaluate the clustering effect without known labels, which is calculated as^39,40^

6 $$SC=\frac{1}{n}\sum_{i=1}^{n}\frac{{b}_{i}-{a}_{i}}{{\mathrm{max}({a}_{i},b}_{i})},$$

where *a*_*i*_ is the average distance between the *i*th sample and other samples in the same group, and *b*_*i*_ is the average distance between the *i*th sample and samples in different groups. A value of *SC* closer to 1 indicates better clustering results, because there is a large distance between samples in different groups, and a small distance between samples in the same group.

Based on above method, the field collected samples are divided into *k* groups, the rock mass parameters in the same group are always similar. In this paper, range expressed in percentiles is used as the evaluated results of the new data belong to each group. For example, *a*th and *b*th percentile is selected as the evaluated results, and the *a*th and *b*th percentile values of each group can be obtained based on the clustering results. Among them, *a* and *b* are ranged from 0 to 100, and *a*th percentile means in each group, there are *a*% samples whose rock mass parameters are lower than it. According to the real-time tunneling parameters, it is easily to judge what group the new-encountered condition belongs to. Assuming the new-encountered condition belongs to the *i*th group, the corresponding rock mass parameter is evaluated to be $\left[{r}_{i}^{a},{r}_{i}^{b}\right]$, where ${r}_{i}^{a}$ and ${r}_{i}^{b}$ represent the *a*th and *b*th percentile values of the *i*th group, respectively. Especially, the value of *a* and *b* is not constant, they should be selected according to actual requirement. The more the range between *a* and *b*, the rougher the evaluation ranges, and the possibility of the located rock parameters in the evaluated ranges.

## Case study

### Project overview and data preparation

**Figure 2:**
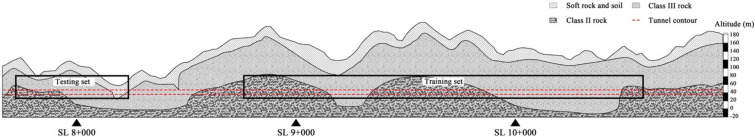
Part of the geological profile.

This research is based on the C1 part of the Pearl Delta water resources allocation project. This area is located in Dongguan, Guangdong Province, China, from Shaxi reservoir to the SL02# work well in Yangwu country. The tunnel goes from west to east. The 2# main cave was excavated by TBM, with a total length of 9.75 km and the diameter of 8.2 m, from mileage SL14 + 958 to SL5 + 213. The inner diameter of the water flow section ranges from 6600 to 8000 mm, and the designed flow velocity is about 2.1 m s^−1^. The burial depth ranges from 13 to 235 m, and the elevation of the tunnel bottom plate ranges 30.9 to 36.1 m. Low mountain and hilly is the main landform along the tunnel. The depth of the tunnel ranges from 50 to 270 m, and it goes under multiple reservoirs and highways. This area is totally high in the east, and the tunnel slope ranges from 20° to 30°. According to the hydropower classification (HC) method, the surrounding rock along the tunnel mainly consists of class II and III rock. Part of the geological profile of the project is shown in Fig. 2.

The tunnel goes through a stratum with multiple lithologies, and the rock condition of different areas changes sharply. Along the tunnel, 130 samples were collected in different strata, and the UCS, and Jf, and corresponding TBM operating parameters of each sample were measured or tested in the field. Among the 130 samples, 100 samples are collected from mileage SL 10 + 560 to SL 8 + 780 and used to obtain the evaluated results by the proposed method as a training set. To verify the method and its results, the other 30 samples were collected from mileage SL 8 + 240 to SL 7 + 810 and consisted of the test set.

Each sample consists of rock mass parameters and tunneling data collected from the same location, among them, the tunneling data are used to cluster samples into groups, and the statistic results of the rock mass parameters distribution are used as the evaluation results of each group. All excavation data are captured from the acquirement system equipped by TBM. The TBM tunneling data were recorded from July 20 2021 to January 10 2022, with a frequency of 1 s-^−1^, including 276 features. More than 15.2 × 10^6^ records were collected.

**Figure 3:**
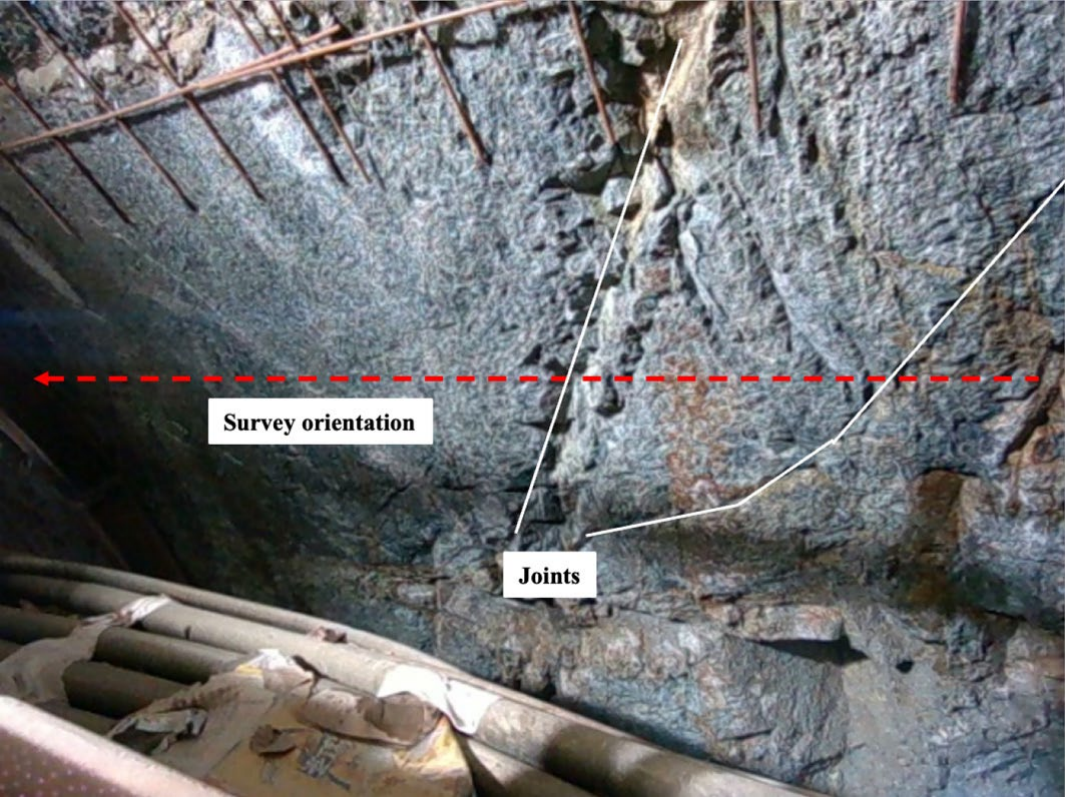
Counting method for joint frequency.

In addition, UCS and Jf is collected as rock mass parameters and evaluating targets. In detail, UCS is obtained by field coring and laboratory compression testing, while by counting the number of joints on the measuring line along the tunnel excavation direction in per unit length, as Fig. 3 shows. According to the results of the field tests, the strength of rock mass parameters changed from 5.2 to 135.6 MPa. The joint frequency ranged from 1.20 to 4.20 m^−1^. The basic information of the rock mass and main TBM tunneling parameters is listed in Table 1.

**Table 1:** Basic information of main field data.

Type	Parameter (unit)	Maximum	Minimum	Average	Std. deviation
Rock mass parameters	Uniaxial compressive strength (MPa)	135.6	5.2	60.8	29.2
	Joint frequency (m^−1^)	4.2	1.2	2.71	0.89
Main TBM tunneling data	Thrust (kN)	10,214.3	2561.5	5435.2	1532.1
	Torque (kN m)	1824.0	199.3	840.4	332.2
	Penetration rate (mm min^−1^)	82.6	8.1	46.7	17.6
	Revolutions per minute (RPM)	5.8	4.0	5.1	0.39

### Data cleaning

A large value of TBM tunneling data contains a large quantity of redundant data, whose volume does not match that of rock mass data. Therefore, data cleaning is conducted to reduce redundancy and match TBM tunneling data to rock mass data. For the purposes of this paper, the cleaned TBM tunneling data should meet three requirements:

1. The retained TBM tunneling data should be collected from or near the same area as the rock mass data and in a limited range, and it is helpful to avoid sharp changes of the rock condition within this range.
2. The abnormal data should be eliminated as much as possible to minimize the negative influence on the retained data.
3. The retained data should be collected during TBM excavation and not TBM stoppage.

**Figure 4:**
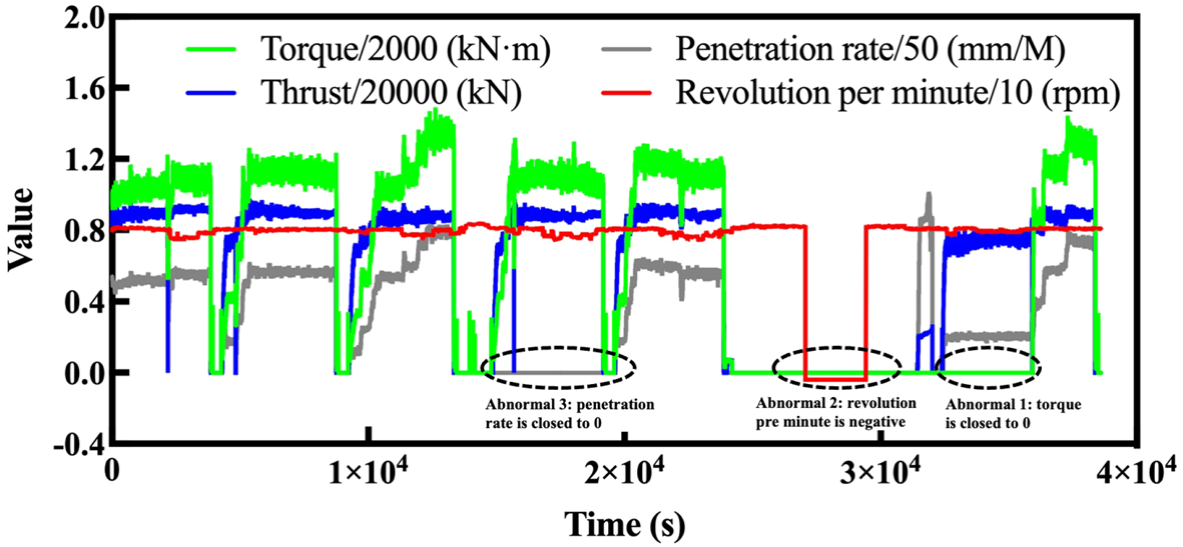
Example of reducing and retaining data.

According to above requirements, we retained the TBM tunneling data collected in the range from behind 0.5 m to forward 0.5 m from the collected location of each rock mass sample. In this way, a long continuous area in which the TBM tunneling data collected were divided into 100 parts with lengths of 1 m, and the rock mass parameters were tested and collected in the center position of each part. This satisfies the first requirement, but each part still includes stoppage data and abnormal data. In order to eliminate the abnormal data, some key tunneling parameters, such as revolution per minute, penetration rate, thrust, and torque are considered as the judgment criteria. In detail, 3 kinds of data are regarded as abnormal data: (1) penetration rate appears normal but the revolution per minute, thrust, or torque is closed to 0 in the same time; (2) data exceeds the critical value or appears abnormal negative values; (3) thrust or torque is continuous normal in long time but the penetration rate is closed to 0. During normal tunneling, the above three situations can hardly occur. Figure 4 shows an example of identifying and cleaning abnormal data. In order to eliminate the stoppage data, we use the penetration rate, i.e., the driving speed of a TBM, as the criterion for judging TBM excavation or stoppage. If a series of TBM tunneling data have penetration rates lower than 10 mm min^−1^, the data are regarded to have been collected during TBM stoppage or trial driving, and this is reduced, while data with penetration rates higher than 10 mm min^−1^ are retained.

After redundant tunneling data reduction, rock mass data and valid TBM tunneling data collected in the matched 100 areas are obtained. However, there is only one series of rock mass data in each 1-m part, while there are usually more than 1000 series of valid tunneling data, because it is sequential with a frequency of 1 s^−1^, and tunneling 1-m-long area always takes more than 1000 s. In the proposed method, each tunneling parameter in a 1-m-long part is used as a feature of sample, which should be a concrete value instead of sequential data including more than one value. Therefore, the average value is used to represent the sequential tunneling data of each, i.e.,

7 $${x}_{ij}^{\prime}=\frac{1}{n}\sum_{k=1}^{n}{x}_{ijk}$$

where ${x}_{ij}^{\prime}$ is the handle results of the *j*th feature of the *i*th sample, while *x*_*ijk*_ represents the value of *x*_*ij*_ measured at the *k*th second, and *n* is the total number of seconds spent in the *i*th part.

## Results

On the basis of the field data, the proposed method with three steps was used, 100 samples were divided into several groups, and we present to demonstrate the efficiency of the proposed method.

### Results of normalization and dimension reduction

**Figure 5:**
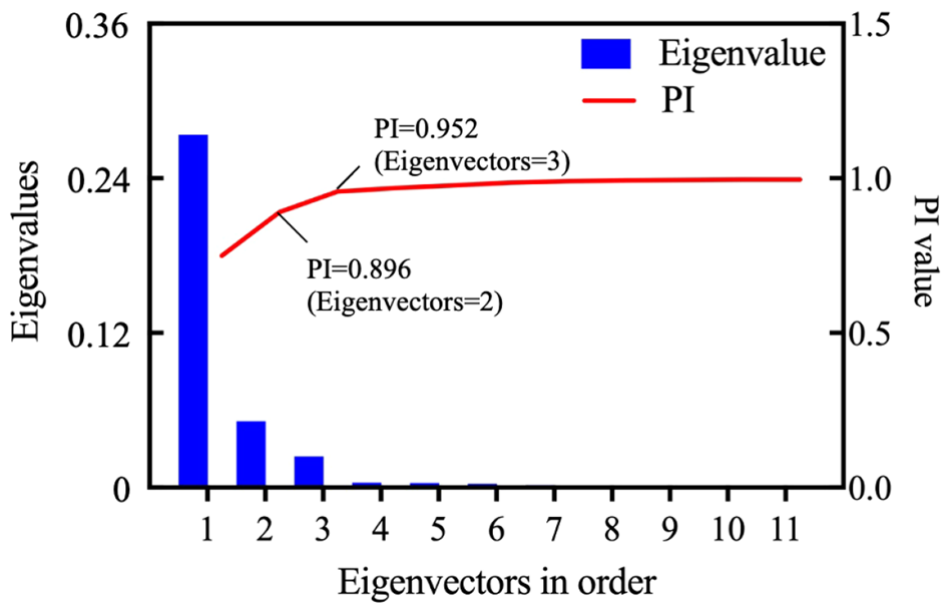
Eigenvalues and their proportion of accumulated information of each handled feature by proposed method.

Through zero-mean normalization, the features of the 100 samples, including rock mass and TBM tunneling parameters, are changed to the range [− 1,1]. Then, logistic normalization is used to bring nonlinear changes to the data, which will change the data distribution and relationship. Then, PCA is used for dimensionality reduction to reduce the irrelevant features. In this procedure, original features are transformed into several newly created handled features, that are ordered from high to low according to the amount of information. To reduce the irrelevant features, the number of handled features to be retained should be determined. Too many retained features may lose the effect of dimensionality reduction, while too few may lead to the loss of key information of the original data. According to the principle of PCA, the handled features are the eigenvectors of the covariance matrix of original features, and the corresponding eigenvalues characterize the information of the handled features. In other words, the larger the eigenvalue, the more information is included in the corresponding eigenvector. Therefore, the proportion of accumulated information (*PI*) is used to determine the number of handled features, and can be calculated by as^40^

8 $${PI}_{l}=\frac{\sum_{i=1}^{l}{k}_{i}}{\sum_{i=1}^{n}{k}_{i}},$$

where *PI*_*l*_ is the proportion of accumulated information from the 1st to *l*th handled features, and *k*_*i*_ represents the eigenvalues of the *i*th handled features, and *n* is the dimension of the covariance matrix, and also the total number of original features. According to Eq. (8), the eigenvalues of each handled feature and the proportion of accumulated information are calculated, and are shown in Fig. 5.

PCA transformed TBM tunneling data include 276 features, and the same number of corresponding eigenvectors are obtained. The results show that, except for the first 11 features, all of the eigenvalues of the handled features are less than 1, these results are not shown in Fig. 5. Usually, as few handled features as possible should be reserved, and the *PI* value should be higher than 0.95^30^. As Fig. 5 shows, only three features with the highest eigenvalues should be reserved, and the dimension of the tunneling data is reduced from 276 to 3. This result means using the 3 new features replaced the original tunneling data with 276 features can retained more than 95% original data information. This shows that this method is effectively simplifies the TBM tunneling data, and provides a processable dataset for clustering.

### Results of k-means clustering

**Figure 6:**
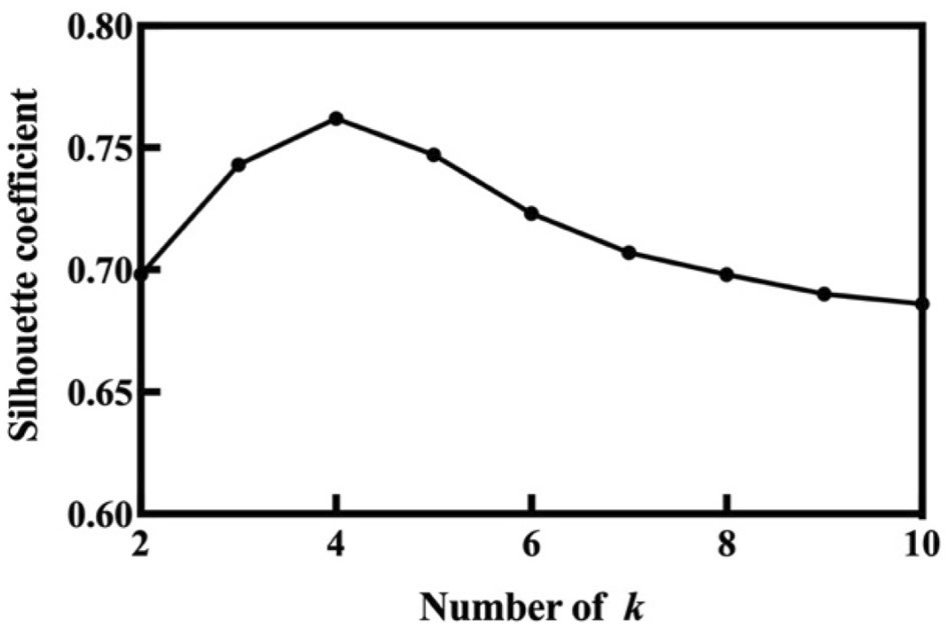
Maximum silhouette coefficient of the k value ranges from 2 to 10.

By normalization and dimension reduction, the number of TBM tunneling data features is reduced to three. According to the three handled features, the 100 samples in the training set which represent 100 different locations of the tunnel are divided into several groups by K-means clustering method. Before conducting K-means, the number of the clusters *k* must be determined. Under limited calculation condition, different value of *k* may lead to the results falling into local optima, and convergence to different clustering results. Usually, the SC is used to evaluate the clustering effect of K-means. Therefore, the SCs under *k* values from 2 to 10 are tested with randomly selected initial centroids among the 100 training samples. To avoid local optimization, 5 times K-means are conducted with different random initial centroids for each *k* value, and the maximum of their SCs is used to judge the rationality of *k*. The maximum SC of each *k* value is shown in Fig. 6, from which we see that the SC reaches its maximum of 0.766 when *k* is selected as 4.

**Figure 7:**
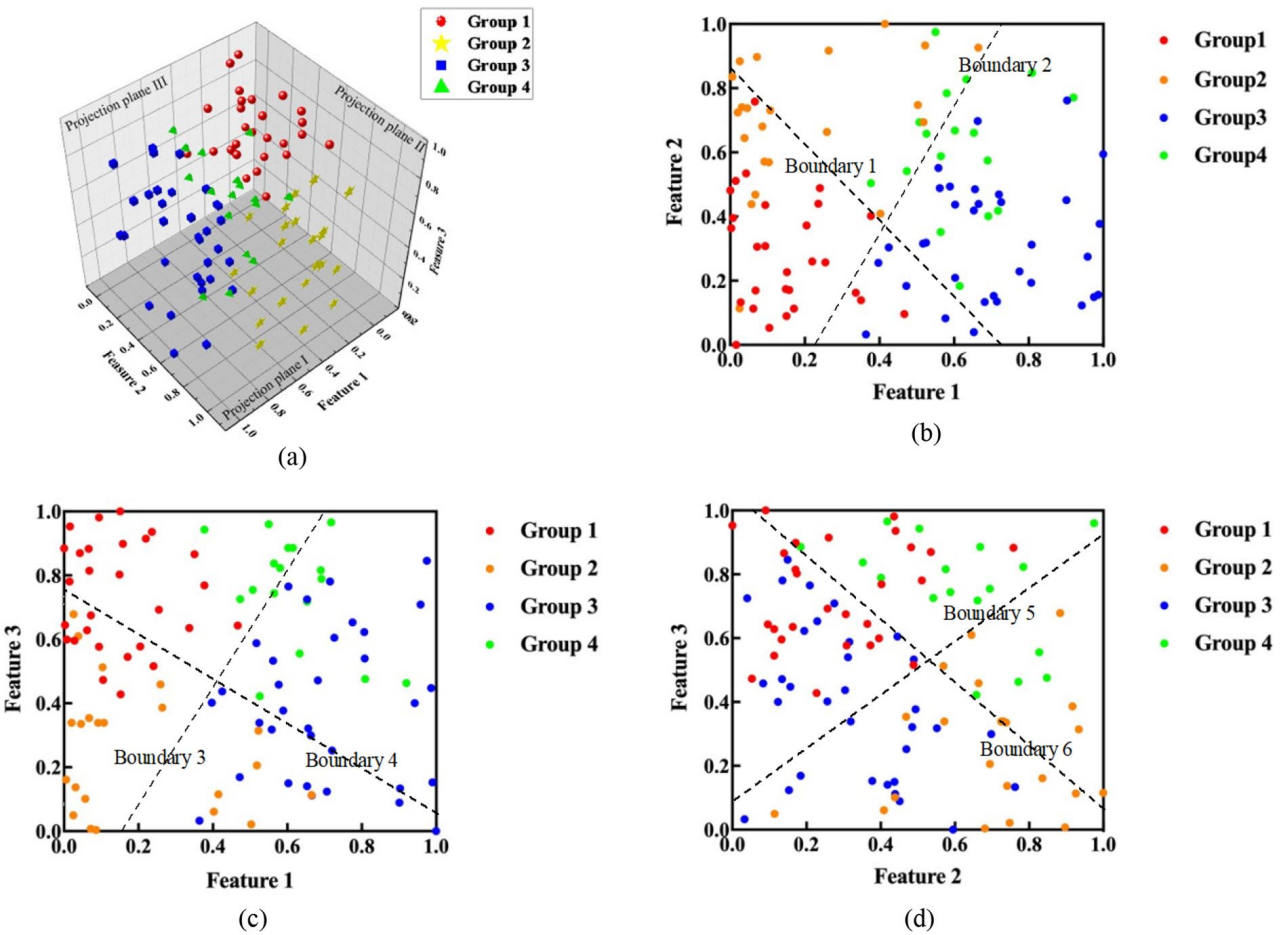
Clustering results of the 100 in-field samples. (**a**) 3D view (**b**) projection on plane I (**c**) projection on plane II (**d**) projection on plane III.

Therefore, the 100 training samples are divided into four groups. Through several iterations, the centroids are updated until the centroids and clustering results remain stable. The centroid location updating process is shown in Table 2. The clustering results are shown in Fig. 7 in 3D and 2D vision.

**Table 2:** Centroid locations in each iteration.

	Centroid 1			Centroid 2			Centroid 3			Centroid 4		
	Feature 1	Feature 2	Feature 3	Feature 1	Feature 2	Feature 3	Feature 1	Feature 2	Feature 3	Feature 1	Feature 2	Feature 3
Iteration 1	0.2094	0.3563	0.6351	0.1950	0.7871	0.1216	0.5219	0.2831	0.5277	0.7841	0.4920	0.8326
Iteration 2	0.1969	0.3497	0.6472	0.1834	0.7753	0.1408	0.5517	0.2955	0.5024	0.7754	0.4981	0.8279
Iteration 3	0.1799	0.3409	0.6637	0.1686	0.7600	0.1656	0.5891	0.3100	0.4705	0.7613	0.5070	0.8230
Iteration 4	0.1626	0.3312	0.6818	0.1560	0.7431	0.1923	0.6260	0.3225	0.4387	0.7406	0.5208	0.8165
Iteration 5	0.1513	0.3214	0.6977	0.1524	0.7268	0.2160	0.6547	0.3295	0.4151	0.7139	0.5408	0.8057
Iteration 6	0.1481	0.3107	0.7108	0.1598	0.7125	0.2345	0.6746	0.3306	0.4019	0.6817	0.5661	0.7893
Iteration 7	0.1495	0.2980	0.7232	0.1756	0.7019	0.2481	0.6889	0.3281	0.3962	0.6467	0.5924	0.7696
**Iteration 8**	**0.1480**	**0.2841**	**0.7353**	**0.1967**	**0.6967**	**0.2567**	**0.6993**	**0.3247**	**0.3939**	**0.6162**	**0.6147**	**0.7509**
Iteration 9	0.1480	0.2841	0.7353	0.1967	0.6967	0.2567	0.6993	0.3247	0.3939	0.6162	0.6147	0.7509
Iteration 10	0.1480	0.2841	0.7353	0.1967	0.6967	0.2567	0.6993	0.3247	0.3939	0.6162	0.6147	0.7509

Significant values are in [bold].

After eight iterations, the centroids remain stable, as Table 2 shows, while the 100 training samples are divided into four groups.

Figure 7a shows the distribution of the four group training samples. The axes in the three orthogonal directions represent the three features after dimension reduction of the tunneling data by PCA, with values ranging from 0 to 1. Although several samples in different groups are distributed closely in the 3D vision, overall, samples in each group have a centralized distribution, and the groups show significant differences in distribution. For the convenience of observation, three orthogonal projection planes are selected. The distribution of the four group samples projected on the planes are shown in Fig. 7a. In 2D vision, distributions of different group samples still have a partial overlap, but samples in any two different groups can be found to have a boundary. Specifically, the above-mentioned boundaries are manually delineated to facilitate intuitive observation of clustering effect. For example, in Fig. 7b, all of the samples in group 1 are distributed left of boundary 1, while all of the samples of group 4 are located to the right of it, which means there are obvious distribution differences between the two groups. Boundaries 2–6 are the boundaries between groups 2 and 3, 1 and 3, 2 and 4, 1 and 2, and 3 and 4, respectively. To sum up, distributions of samples in any two groups have significant differences, which demonstrates acceptable clustering results.

### Evaluated results of rock mass parameters

**Figure 8:**
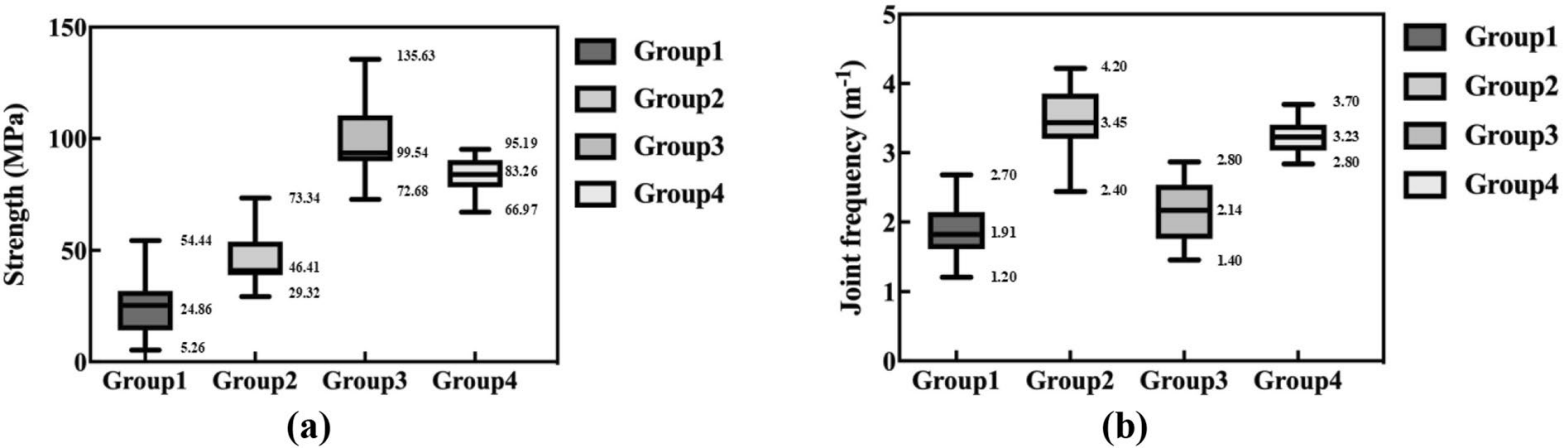
Ranges of rock mass parameters of each group. (**a**) Distribution of uniaxial compress strength (**b**) distribution of joint frequency.

From the statistics rules of the field TBM tunneling data, the 100 samples are divided into four groups by K-means clustering. As "Results of k-means clustering" shows, the clustering results have enough differences between groups and similarity between same-group samples. To evaluate the rock mass parameters on the basis of the known tunneling data, the distribution of the rock mass parameters of the 100 samples is shown in Fig. 8.

As Fig. 8 shows, the rock mass parameter distribution has differences between samples in different groups to varying degrees. From the perspective of all rock mass parameters, there are obvious differences among various group samples, which is helpful for evaluating rock mass parameters in different groups.

To avoid the negative influence of partial outliers, the ranges between the *a*th and *b*th percentile values are regarded as the evaluation results of each group, while *a* and *b* are not constant and selected based on actual requirement. This paper takes the ranges between 25 and 75th percentile values as an example, to analyze the feasibility of evaluation results. Assuming the current tunneling data conform to the characteristics of group 1, the corresponding uniaxial compressive strength, and joint frequency are evaluated to be in the ranges of [14.72, 31.93] MPa, and [1.61, 2.33] m^−1^, respectively. The evaluation results of the four groups of rock mass parameters are listed in Table 3.

**Table 3:** Evaluated ranges of rock mass parameters.

	Uniaxial compressive strength (MPa)		Joint frequency (m^−1^)	
	Lower bound (25th percentile)	Upper bound (75th percentile)	Lower bound (25th percentile)	Upper bound (75th percentile)
Group 1	14.72	31.93	1.61	2.33
Group 2	38.62	57.70	3.15	3.87
Group 3	89.93	110.98	1.76	2.54
Group 4	73.85	92.43	2.94	3.48

**Figure 9:**
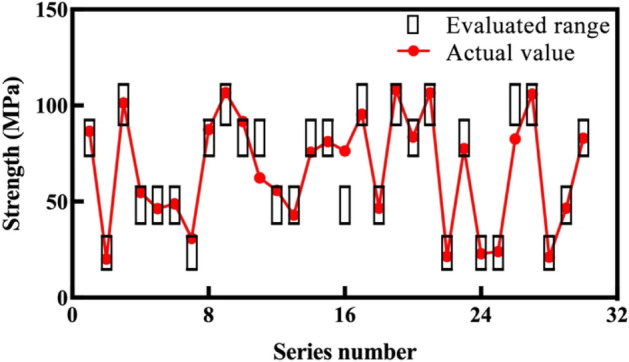
Comparison results of uniaxial compressive strength.

**Figure 10:**
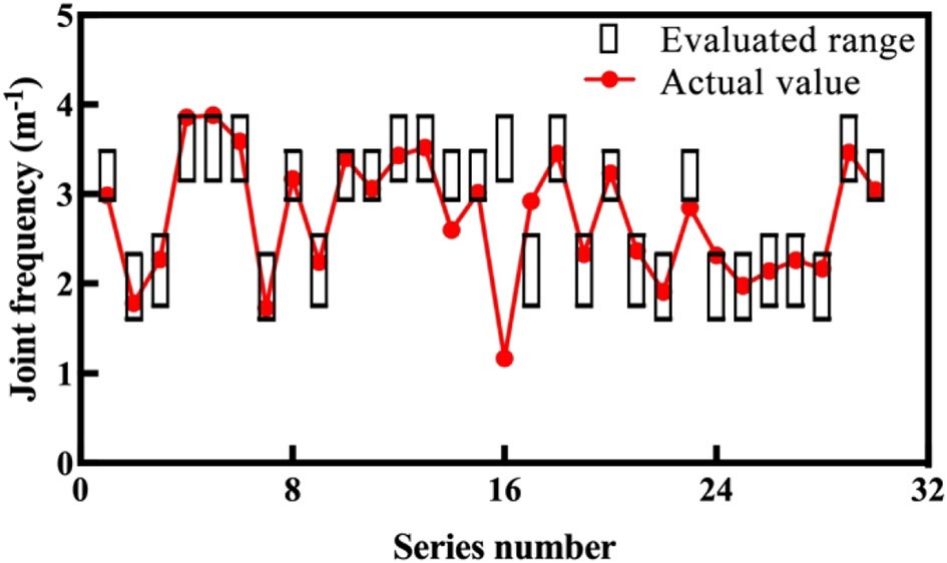
Comparison results of joint frequency.

To confirm the practicability of the proposed evaluation method, 30 samples of the testing dataset are evaluated. Their tunneling data are normalized, their dimensions are reduced, and the normalized distance between each sample and centroid are shown in the last row of Table 2. According to the distance, the tested 30 samples are divided into four groups, and the corresponding rock mass parameter evaluation results are obtained. The comparison results of the measured and evaluated rock mass parameters are shown in Figs. 9 and 10.

Figures 9 and 10 show the comparison results between the evaluated ranges (black boxes) and actual values (red plots) of the mentioned rock mass parameters. For each parameter, only four kinds of evaluated range are given, which correspond to the four groups. The actual rock mass parameters of most samples are located in the evaluated ranges, which can be regarded as the correct evaluation. The actual values of only three uniaxial compressive strength samples (11th, 16th, and 26th), and four joint frequency samples (14th, 16th, 17th, and 23rd) are located outside the black boxes, which is not a correct evaluation. The evaluated accuracies by the proposed method of the two rock mass parameters are 90%, and 86.7%, which is relatively acceptable.

## Discussion

### Verification of normalization and dimension reduction method

**Figure 11:**
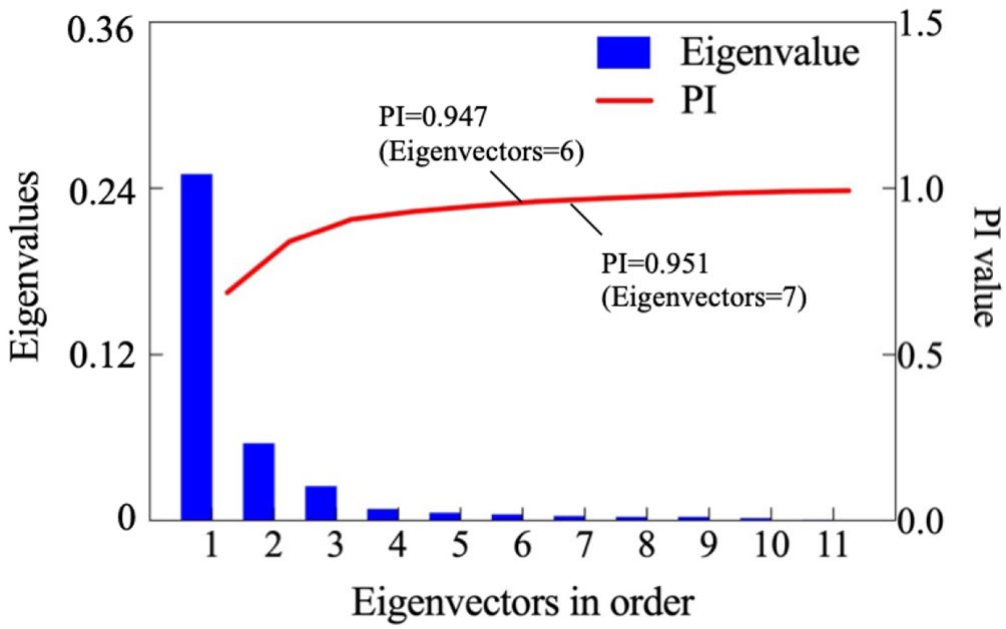
Eigenvalues and their proportion of accumulated information of each handled feature by the zero-mean normalization.

To simplify the massive heterogeneous tunneling data, the PCA algorithm with ensemble normalization is used to reduce the data dimension. However, we must still verify that these methods are effective and superior still needs to be verified. We do this with the zero-mean and t-distributed stochastic neighbor embedding (t-SNE) algorithms, respectively, which are also widely used in solving complex data. The pure zero-mean normalized features are handled by PCA, and the corresponding eigenvalues and their proportion of accumulated information of each handled feature are calculated and shown in Fig. 11, and are used to evaluate the iterative efficiency. In this paper, the effect of normalization and dimension reduction is considered as integrated, and it is judged by the value of *PI*. Provided that the PI value is greater than 0.95, the method of retaining fewer features is better.

According to the PCA method, as few handled features as possible are reserved under the *PI* value higher than 0.95. As Fig. 11 shows, six features handled only by zero-mean normalization should be reserved, the *PI* value reaches 0.960, and the proportions of the first three eigenvalues in the sum are 0.686, 0.153, and 0.068, respectively. After involving the nonlinear changes by logistic normalization, only three features are reserved, the *PI* value reaches 0.957, and the proportion of the first eigenvalues are 0.750, 0.141, and 0.066. The results show that the information content of the features handled by zero-mean and logistic normalization are more concentrated, and it benefits the dimension reduction process. In fact, only three features handled by the integrated normalization method are reserved and the *PI* value exceed the acceptable level (0.95). Compared with the single zero-mean values, integrated normalization can save calculation resources and improve the calculational efficiency of the clustering process.

To verify the superiority of PCA, the t-SNE algorithm is used to solve the heterogeneous tunneling data samples. Different from PCA, the dimension numbers of t-SNE are manually chosen before calculation. We choose this as 3, which is consistent with the results of PCA. The objective function of t-SNE is^41^

9 $$C=\sum_{i}\sum_{j}{p}_{ij}{\mathrm{log}}_{2}\frac{{p}_{ij}}{{q}_{ij}},$$

where *p*_*ij*_ and *q*_*ij*_ are the respective joint probabilities of the original data (in the high-dimension space) and dimension-reduced data of the *i*th and *j*th samples, i.e., *x*_*i*_ and *x*_*j*_, which are influenced by the distance between *x*_*i*_ and *x*_*j*_. The greater the distance the larger the values of *p*_*ij*_ and *q*_*ij*_, which are calculated as^41^

10 $${p}_{j|i}=\frac{exp\left(-1/2{\Vert {x}_{i}-{x}_{j}\Vert }^{2}/{\sigma }_{i}^{2}\right)}{{\sum }_{k\ne i}exp\left(-1/2{\Vert {x}_{i}-{x}_{k}\Vert }^{2}/{\sigma }_{i}^{2}\right)},$$

11 $${p}_{ij}=\frac{1}{2}\left({p}_{i|j}+{p}_{j|i}\right),$$

12 $${q}_{j|i}=\frac{{\left(1+{\Vert {y}_{i}-{y}_{j}\Vert }^{2}\right)}^{-1}}{{\sum }_{k\ne i}{\left(1+{\Vert {y}_{k}-{y}_{i}\Vert }^{2}\right)}^{-1}},$$

where *x* and *y* are the original and dimension-reduced data, respectively. Before t-SNE is executed, an initial *y* is randomly generated, and *C* decreases iteratively by gradient descent^41^. To unify the comparison criteria with PCA, the information lost during dimension reduction should be calculated, which can be expressed by the objective function *C*. Before iteration, the dimension-reduced data are selected randomly, and the initial data information is totally lost. At this time, the information lost is recorded as 1, and *C* reaches its maximum. During the continuous iterative reduction of *C*, the distribution of the dimension-reduced data is getting closer to that of the initial data, and the information lost is reduced. Based on above recognition, the ratio between *C* and its maximum is used to express the information lost, i.e.,

13 $$LOSS=\frac{C}{{C}_{max}}.$$

**Figure 12:**
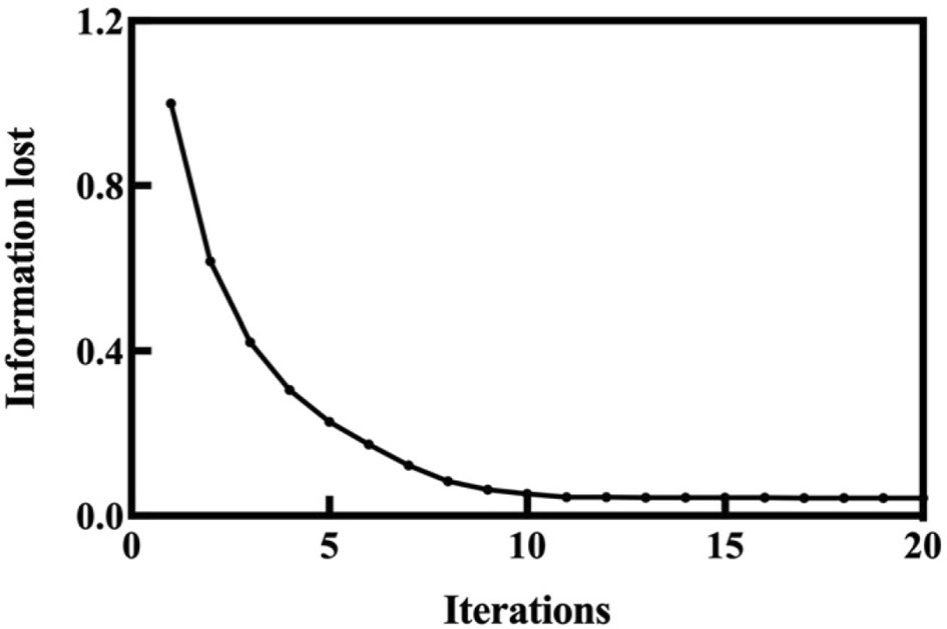
Information lost during t-SNE iteration.

The changes of information lost during iteration are shown in Fig. 12.

A total of 20 iterations occur, after which the information lost reaches 0.053, and remains almost stable, i.e., about 94.7% of the information of the original tunneling data is retained. The result shows that the t-SNE method can retain the information of the original data relatively completely, and the distribution of the dimension-reduced data can well characterize the original tunneling data. Eleven iterations are used by t-SNE to reduce the information lost to near 0.05, but still somewhat larger than that (Fig. 12). To sum up, the t-SNE method achieves acceptable results, which are close to those of PCA. However, for the task of solving tunneling data, PCA still has a tiny advantage regarding the loss of data information. In addition, PCA has no iteration, which makes the method simpler and more convenient.

### Verification of clustering method

We verify the effectiveness and superiority of K-means by comparing it with other clustering methods that can be used to reach a similar goal. Many researchers have used fuzzy C-means to solve TBM-related clustering problems^42–44^ which is inspiring for this research. Different from K-means, fuzzy C-means uses membership of each sample to each cluster to represent the results, instead of dividing samples into specific clusters. For verification, fuzzy C-means is used on the same 100 field samples, and the results are compared with those of K-means.

Before executing fuzzy C-means, the number of clusters is selected. To maintain consistency with *K-*means, the number of clusters is also selected to be 4. By minimizing the objective function (Eq. 14) during iteration, the centroids of each group can be calculated. Taking the task of dividing *N* samples into *K* groups as an example, the objective functions can be expressed as

14 $$J=\sum_{i=1}^{K}{J}_{i}=\sum_{i=1}^{K}\left(\sum_{j=1}^{N}{{w}_{ij}}^{m}{\Vert {X}_{j}-{C}_{i}\Vert }^{2}\right),$$

where *X*_*j*_ represents the data of the *j*th sample; *C*_*i*_ is the centroid of the *i*th group; *w*_*ij*_ is the weight, which ranges from 0 to 1, and is randomly generated before iteration; and *m* is a fuzzification parameter that is larger than 1. The sum of weights belonging to the same sample is 1, i.e.,

15 $$\sum_{i=1}^{K}{w}_{ij}=1.$$

The centroids of the *K* groups can be calculated as.

16 $${C}_{i}=\frac{\sum_{j=1}^{N}{{w}_{ij}}^{m}{X}_{j}}{\sum_{j=1}^{N}{{w}_{ij}}^{m}},$$

given a known weight matrix. When the centroids are obtained, the weight matrix can be updated by.

17 $${w}_{ij}=\frac{1}{\sum_{s=1}^{K}{\left(\frac{\Vert {X}_{j}-{C}_{i}\Vert }{\Vert {X}_{j}-{C}_{s}\Vert }\right)}^{\frac{2}{m-1}}}.$$

During multiple iterations, the objective function value is continuously decreasing, and iterations continue until each reduction is less than a specific value. For sufficient iterations to avoid local optimization, a small value of 0.01 is selected in this paper.

Fuzzy-C means clustering generally uses a weight matrix to express the results. However, the proposed evaluation method needs hard clustering, i.e., samples should be clearly divided into groups. Only in this way can the rock mass parameter distribution of each group be obtained. Therefore, a hardening process is applied to the general fuzzy-C clustering method. Each sample has four membership degree values corresponding to the four groups, a sample is clustered into a group that has a maximum degree value.

Using fuzzy-C means clustering, the 100 training samples are clustered into four groups, which is different from the results of K-means. The 25th and 75th percentile values of each rock mass parameters are listed in Table 4, and are regarded as the evaluated ranges by fuzzy-C means clustering.

**Table 4:** New evaluated ranges by fuzzy c-means.

	Uniaxial compressive strength (MPa)		Joint frequency (m^−1^)	
	Lower bound (25th percentile)	Upper bound (75th percentile)	Lower bound (25th percentile)	Upper bound (75th percentile)
Group 1	14.71	35.17	1.60	2.15
Group 2	36.63	52.70	3.37	3.85
Group 3	73.65	106.22	2.19	3.08
Group 4	80.47	109.63	1.77	3.20

**Figure 13:**
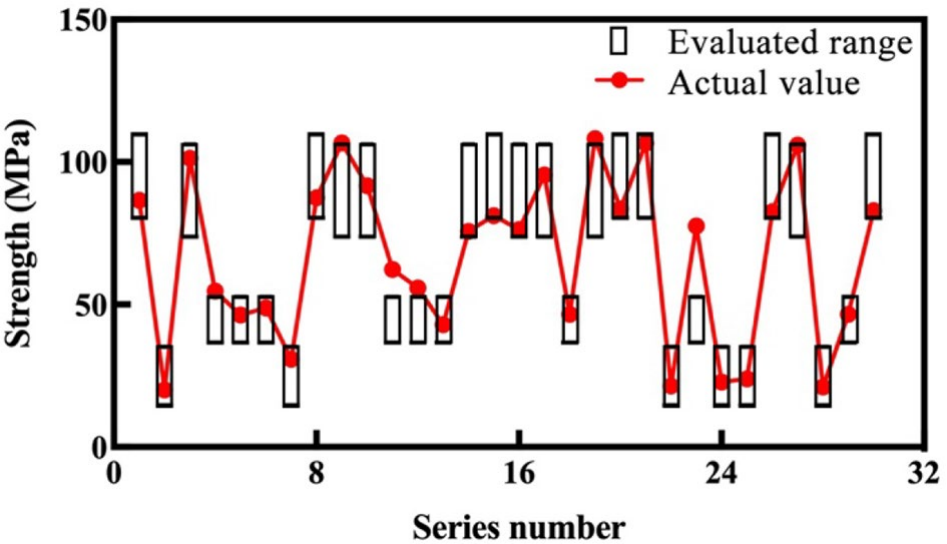
Evaluated results of the uniaxial compressive strength by fuzzy C-means.

**Figure 14:**
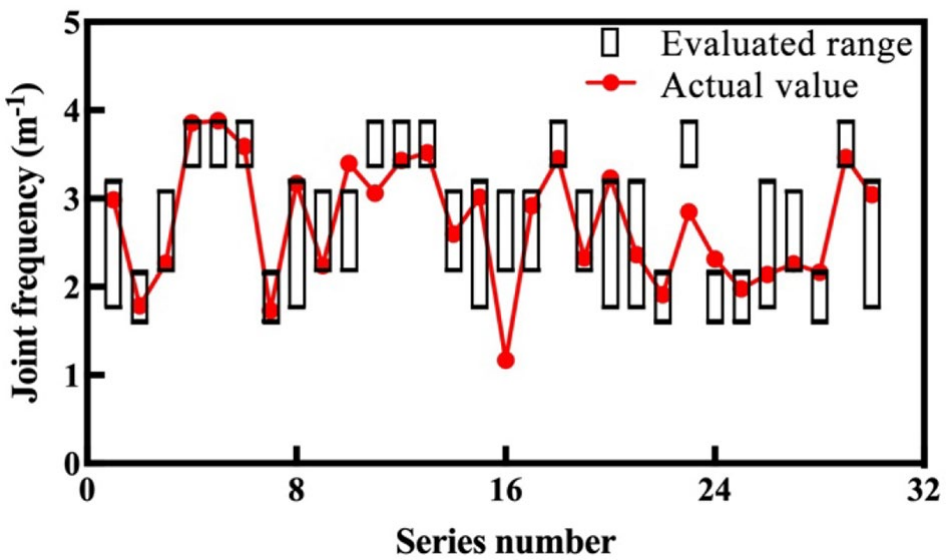
Evaluated results of the joint frequency by fuzzy C-means.

The new evaluated ranges by fuzzy C-means are also verified by the same 30 testing samples, that are used to test the ranges generated using the K-means clustering method. The evaluated results by the fuzzy C-means and K-means are used to verify the proposed method. The results of fuzzy C-means are shown in Figs. 13 and 14.

Figures 13 and 4 show that fuzzy C-means also has acceptable results, with only five uniaxial compressive strength samples (4th, 11th, 12th 19th, and 23rd), and five joint frequency samples (10th, 11th, 16th, 23rd, and 24th) with incorrect evaluations. The fuzzy C-means accuracy of the two rock mass parameters is 83.3%. On the whole, K-means is more accurate than fuzzy C-means.

### Limitation

In this paper, a method to evaluate rock mass parameters based on known TBM tunneling data is proposed. Although the method has been proved to be reasonable by the field data collected in the C1 part of the Pearl Delta water resources allocation project, there are still several limitations of the method.

1. One of key innovations in this paper is solving the volume and structure difference between rock mass parameters and TBM tunneling data. In addition to volume and structure, the abnormal data also has significant negative influence on evaluation results. In case study process, this research used 3 simple criteria to judge if the data is abnormal, so that computer algorithms can be used to clean data rapidly. However, TBM tunneling data is complex, this method can only remove partly simple and obvious abnormal not all. Therefore, a small amount of abnormal data inevitably limits this method, and useful data cleaning method should be further studied.
2. The proposed method has successfully clustered samples into several different groups. Considering that the rock mass parameters of samples in each group has similar distribution, the method has the potential to assist in establishing multiple mappings between rock mass parameters and TBM tunneling data to improve the accuracy of single mappings. For this purpose, samples in each group rather than all samples can be used as an independent training set to establish machine learning model. However, after clustering, there are only less than 30 samples in each group, which is not enough for training. Therefore, more quantities and types of samples should be collected in further investigation, and method for building multiple mappings also needs further research.
3. The method proposed in this paper is originated from field-collected data and has been verified to be effective. However, if the method is applicated to different project still needs to be studied. Therefore, the method is recommended to be used for similar rock mass conditions, i.e. rock of class II or III according to the hydropower classification (HC) method. Further, the samples used for training is collected in the area with relatively integrity rock, with the joint frequency lower than 4.2 m^−1^. For different rock condition from this project, especially broken rock condition, more field-collected data is needed in further research.

## Conclusion

We introduced a method to solve TBM tunneling parameters based on K-means clustering. Using this method, target stratum samples are divided into several groups and evaluated according to their differences of TBM tunneling parameters. The proposed method was realized by 100 training samples and verified by 30 testing samples collected from the C1 part of the Pearl Delta water resources allocation project. Our main conclusions are summarized as follows:

1. Different from a traditional single normalization method, logistic normalization is conducted after zero-mean normalization to bring nonlinear changes to the features, which is shown to be helpful for dimension reduction using in-field data. After single zero-mean normalization, 276 features of TBM tunneling data were reduced to five features by the PCA method, while only three were retained according to the logistic and zero-mean method. It was shown that, using the method proposed in this paper, heterogeneous TBM tunneling data are further simplified to be processable;
2. By randomly selecting sample combinations as initial centroids and eight iterations, 100 training samples were divided into four groups. The SC of the clustering results reached 0.768. In three 2D orthogonal projection planes, boundaries between samples in any two different groups could be found. The distribution of samples in any two different groups had significant differences, which showed that the clustering results were acceptable;
3. Based on the clustering results of 100 training samples, the ranges between the 25th and 75th values of each parameter were regarded as the evaluated results, which was further verified by the 30 testing samples. The evaluated accuracies of the UCS and joint frequency reached 90%, and 86.7%, respectively, which shows that the evaluation results reached an acceptable value, and the proposed method was helpful for judging rock conditions.

## Data Availability

The datasets generated and analyzed during the current study are not publicly available due requirements of our partners, but are available from the corresponding author on reasonable request.
